# PD-L1 confers resistance to EGFR mutation-independent tyrosine kinase inhibitors in non-small cell lung cancer via upregulation of YAP1 expression

**DOI:** 10.18632/oncotarget.23161

**Published:** 2017-12-08

**Authors:** Jai-Nien Tung, Po-Lin Lin, Yao-Chen Wang, De-Wei Wu, Chi-Yi Chen, Huei Lee

**Affiliations:** ^1^ Department of Neurosurgery, Tungs’ Taichung MetroHarbor Hospital, Taichung, Taiwan; ^2^ Graduate Institute of Cancer Biology and Drug Discovery, Taipei Medical University, Taipei, Taiwan; ^3^ Department of Internal Medicine, Chung Shan Medical University and Hospital, Taichung, Taiwan; ^4^ Department of Surgery, Chung Shan Medical University and Hospital, Taichung, Taiwan

**Keywords:** PD-L1, TKI, YAP1, NSCLC

## Abstract

Programmed death ligand (PD-L1) expression was associated with tumor immune escape and subsequent poor prognosis in non-small cell lung cancer (NSCLC). This expression was higher in patients with EGFR-mutated NSCLC tumors than in those with EGFR-wild-type (WT) NSCLC tumors. We therefore hypothesized that poor prognosis mediated by higher PD-L1 may be partially through conferring resistance to tyrosine kinase inhibitor (TKI) in NSCLC regardless of EGFR mutation. The change in PD-L1 expression following gene manipulation corresponded with changes in expression of HIF-1α and YAP1. The expression of HIF-1α and YAP1 was concomitantly decreased by PD-L1 silencing or by ROS scavenger treatment (N-acetylcysteine, NAC); however, a ROS inducer treatment (pyocyanin) completely reversed the decreased expression of both genes in EGFR-mutated and -wild-type (WT) NSCLC cells. The MTT assay indicated that the inhibitory concentration of gefitinib yielding 50% cell viability (IC50) depended on PD-L1-mediated YAP1 expression. Mechanistic studies indicated that upregulation of YAP1 by PD-L1 might be responsible for EGFR mutation-independent TKI resistance via the ROS/HIF-1α axis. An unfavorable TKI response was more common in patient tumors with high PD-L1 or YAP1 mRNA expression than in patient tumors with low mRNA expression of these genes. In conclusion, PD-L1 might confer EGFR mutation-independent TKI resistance in NSCLC cells via upregulation of YAP1 expression.

## INTRODUCTION

The binding of programmed death ligand 1 (PD-L1) to its receptor PD-1 leads to T cell apoptosis, which, in turn, triggers tumor immune surveillance [1, 2]. PD-L1 overexpression has a known association with poor prognosis in various human cancers, including non-small cell lung cancer (NSCLC), due to tumor immune escape [3]. PD-L1 expression is also higher in patients with epidermal growth factor receptor (EGFR)-mutated NSCLC tumors than with EGFR-wild-type (WT) tumors [4, 5]. A higher PD-L1 expression in EGFR-mutated tumors may alter the tumor immune microenvironment, thereby promoting tumors with a more aggressive phenotype and consequently resulting in poor prognosis [4]. Therefore, we predicted that PD-L1 might make a significantly greater contribution to tumor malignancy in patients with EGFR-mutated NSCLC tumors than with EGFR-WT tumors.

Tumor metastasis and drug resistance represent a crucial limitation in human cancer therapy. EGFR-tyrosine kinase inhibitors (EGFR-TKIs) have known clinical benefits in the treatment of EGFR-mutated NSCLC when compared to EGFR-WT NSCLC [6, 7]. Unfortunately, resistance to TKIs frequently arises in about 9 to 12 months in patients undergoing TKI treatment, and consequently to result in patients’ tumor relapse and death [8–10]. Previously, reduction of FOXO3a, microRNA-200c expression and overexpression of insulin growth factor 1 receptor and phosphorylated EGFR were associated with worse efficacy of EGFR-TKIs in NSCLC patients with EGFR-WT [11–14]. However, the TKI resistance in EGFR-WT NSCLC is still largely unclear. Therefore, discovery of possible mechanisms in EGFR mutation-independent TKI resistance may not only improve TKI resistance in EGFR-mutated NSCLC but also in EGFR-WT NSCLC.

EGFR-TKIs down-regulate PD-L1 expression in EGFR-mutated NSCLC through inhibition of the NF-κB signaling pathway [15]. PD-L1 expression is also downregulated by inhibitors of the MEK/ERK and PI3K/AKT signaling pathways in EGFR-mutated NSCLC cells [16–18]. The decrease in PD-L1 expression by these inhibitors may suppress tumor progression by altering tumor immune surveillance. On the other hand, PD-L1 expression was associated with epithelial-to-mesenchymal transition (EMT), high proliferation activity, and poor prognosis in adenocarcinoma of the lung [19–21]. EMT has known to confer TKI resistance in NSCLC [22]. However, the role of PD-L1 in EGFR mutation-independent TKI resistance in NSCLC remains unclear.

The development of the hypoxic tumor microenvironment, which is associated with tumor progression and metastasis, depends on the presence of myeloid-derived suppressor cells (MDSCs) and tumor-associated macrophages (TAMs) [23, 24], and arises due to the generation of reactive oxygen species (ROS) and subsequent induction of HIF-1α expression [25, 26]. PD-L1, as a direct target of HIF-1α, is blocked under hypoxia, while MDSC-mediated T cell activation is enhanced [27]. High ROS levels are also associated with an increase in YAP1 expression [28]. Increases in PD-L1 expression confer cisplatin resistance in small cell lung cancer and NSCLC cells [29, 30]. We therefore hypothesized that PD-L1 expression might confer EGFR mutation-independent TKI resistance.

Our preliminary data showed that PD-L1, HIF-1α, and YAP1 expressed concomitantly in EGFR-mutated and EGFR-WT NSCLC cells. YAP1 confers chemoresistance in NSCLC cells [31], and YAP1 inhibition restores the sensitivity to EGFR-TKI in TKI-resistant lung adenocarcinoma [32]. Here, we provide evidence that YAP1 expression may be responsible for PD-L1-mediated TKI resistance in NSCLC cells regardless of EGFR mutation.

## RESULTS

### PD-L1, HIF-1α, and YAP1 concomitantly expressed, and PD-L1 may confer TKI resistance in EGFR-mutated and EGFR-WT cells

**Figure 1:**
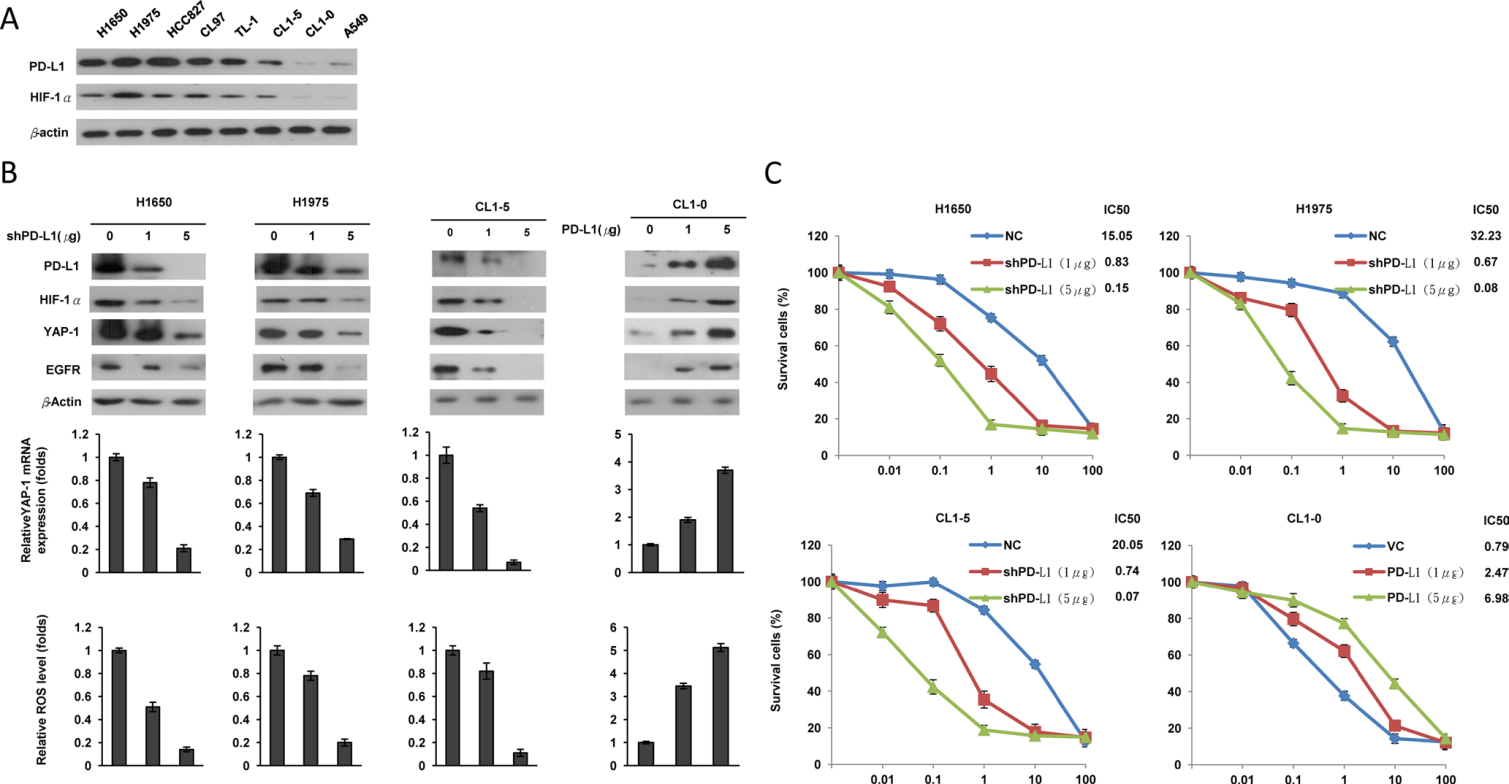
PD-L1, HIF-1α, and YAP1 are concomitantly expressed, and PD-L1 may confer TKI resistance in EGFR-mutated and EGFR-WT cells (**A**) Western blottingnanalysis was performed to evaluate PD-L1 expressions in different lung cancer cell lines. (**B**) H1650, H1975 and CL1-5 cells were transfected with PD-L1 shRNA, while CL1-0 cells were transfected with a PD-L1 expressing plasmid for 48 h. The expression of PD-L1 and YAP1 in these cells was evaluated by western blotting. The mRNA level was determined by real-time PCR. ROS production in response to Cells with transfected with vector control or PD-L1 expression vector/shRNA was determined by a flow cytometry following ROS detection kit. The relative ROS levels are shown in the lower panel. (**C**) Cells were treated with six concentrations of gefitenib to calculate the IC50 value from the dose-response survival curve determined by the MTT assay.

Four EGFR-mutated and EGFR-WT cell types were collected to verify whether PD-L1 expression could be associated with HIF-1α and YAP1 expression. Western blotting indicated a higher PD-L1 expression in EGFR-mutated cells than in EGFR-WT cells (Figure 1A). This expression of PD-L1 was consistently observed together with HIF-1α expression in all tested cell types (Figure 1A). PD-L1 expression was manipulated in three cell types with high and one with low expression of PD-L1 and HIF-1α by the use of a small hairpin (sh)RNA and an expression plasmid. A dose-dependent change in PD-L1 expression observed all four tested cell types (Figure 1B, upper panel). Surprisingly, the expression of HIF-1α and YAP1 decreased and increased concomitantly with the PD-L1 expression by these manipulations (Figure 1B, upper panel). The changes induced in YAP1 protein expression by PD-L1 manipulation were consistent with its mRNA levels (Figure 1B, middle panel), suggesting that modulation of YAP1 expression by PD-L1 might occur at the transcriptional level. In addition, the changes in HIF-1α expression following PD-L1 manipulation correlated with ROS levels in these cells (Figure 1B, lower panel). These results suggest that PD-L1 may increase HIF-1α and YAP1 expression by increasing ROS generation in these cells.

We next examined the possibility that PD-L1 could be responsible for TKI resistance in NSCLC cells. Two EGFR-mutated and EGFR-WT cell types collected for manipulation of PD-L1 expression to test this possibility. The MTT assay indicated that the inhibitory concentration of gefitinib yielding 50% cell viability (IC50) decreased markedly by PD-L1 silencing in H1650, H1975, and CL1-5 cells. The IC50 values were gradually increased by ectopic PD-L1 expression in CL1-0 cells (Figure 1C). These results suggest that PD-L1-mediated TKI resistance might occur by an increase in HIF-1α and YAP1 expression due to enhanced ROS generation.

### PD-L1 may induce HIF-1α by ROS generation, and in turn, upregulate YAP1 expression in NSCLC cells regardless of EGFR mutation

**Figure 2:**
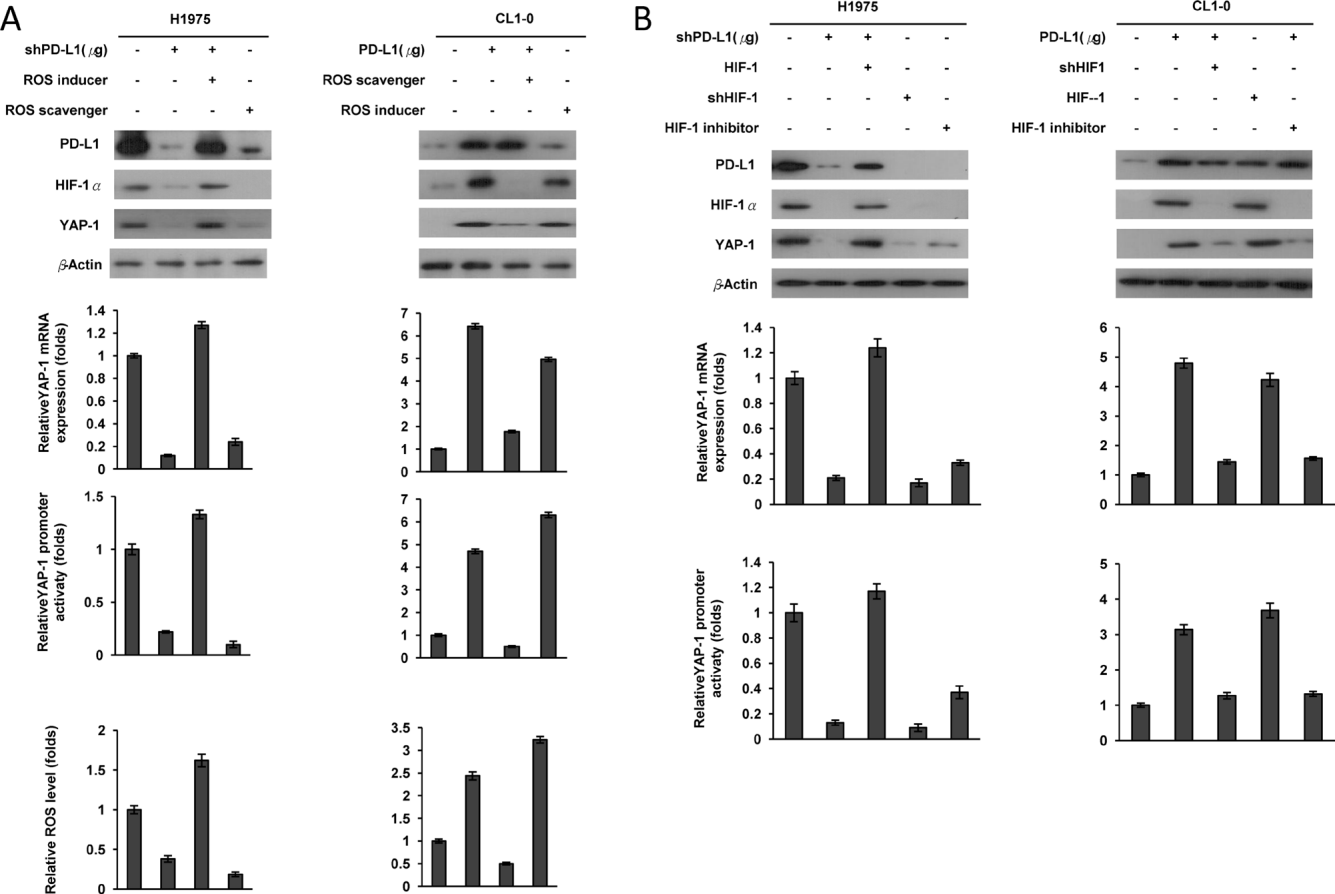
PD-L1 may induce HIF-1α by ROS generation, and in turn, upregulate YAP1 expression in NSCLC cells (**A**) ROS inducer (pyocyanin), ROS scavenger (NAC), PD-L1 plasmid and/or PD-L1 shRNA were treated with H1975 and CL1-0 cells for 12 h. The expression of PD-L1 and YAP1 in these cells was evaluated by western blotting. The mRNA level was determined by real-time PCR. The changes in YAP1 promoter activity were evaluated by the luciferase reporter assay. ROS production in response to cells were determined by a flow cytometry following ROS detection kit. (**B**) H1975 cells were transfected with PD-L1 shRNA, HIF-1 shRNA, HIF-1 expression plasmids or HIF-1 inhibitor; CL1-0 cells were transfected with a PD-L1, HIF-1 expressing plasmids, HIF-1 shRNA or HIF-1 inhibitor for 48 h. The expression of PD-L1, HIF-1, and YAP1 in these cells was evaluated by western blotting using their specific antibodies. The mRNA level was determined by real-time PCR. The changes in YAP1 promoter activity were evaluated by the luciferase reporter assay.

We next examined whether PD-L1 may induce HIF-1α expression through ROS generation, thereby upregulating YAP1 expression at the transcription level. H1975 and CL1-0 cells were collected to manipulate PD-L1 expression and/or treatment with a ROS inducer (pyocyanin) or a ROS scavenger (N-acetylcysteine; NAC). Western blotting showed a concomitant decrease in expression of HIF-1α and YAP1 following shPD-L1 transfection or treatment with NAC. However, the decrease in HIF-1α and YAP1 expression by PD-L1 silencing reversed by pyocyanin treatment in both cell types (Figure 2A, upper panel).

The results from real-time PCR, luciferase reporter assays, and flow cytometry analysis confirmed that PD-L1 expression correlated with HIF-1α expression, with YAP1 mRNA expression and reporter activity, and with ROS levels in both cell types, as determined by PD-L1 manipulation and/or ROS inducer or ROS scavenger treatment (Figure 2A, lower panel). The HIF-1α expression and the YAP1 mRNA expression and reporter activity correlated with the ROS levels generated following PD-L1 manipulation plus ROS inducer or scavenger treatments (Figure 2A, lower panel). We also used H1975 and CL1-0 cells in which we manipulated PD-L1 expression. These cells were transfected with an HIF-1α expression plasmid (shHIF-1α) or treated with an HIF-1α inhibitor (2-methoxyestrodiol) to confirm that PD-L1 induced HIF-1α expression and, in turn, upregulated YAP1 transcription (Figure 2B). These results clearly indicate that PD-L1 may stimulate ROS production, which induces HIF-1α expression, and consequently, upregulates YAP1 transcription in NSCLC cells regardless of EGFR mutation.

### PD-L1-mediated YAP1 expression is responsible for EGFR mutation-indepdendent TKI resistance in NSCLC cells

**Figure 3:**
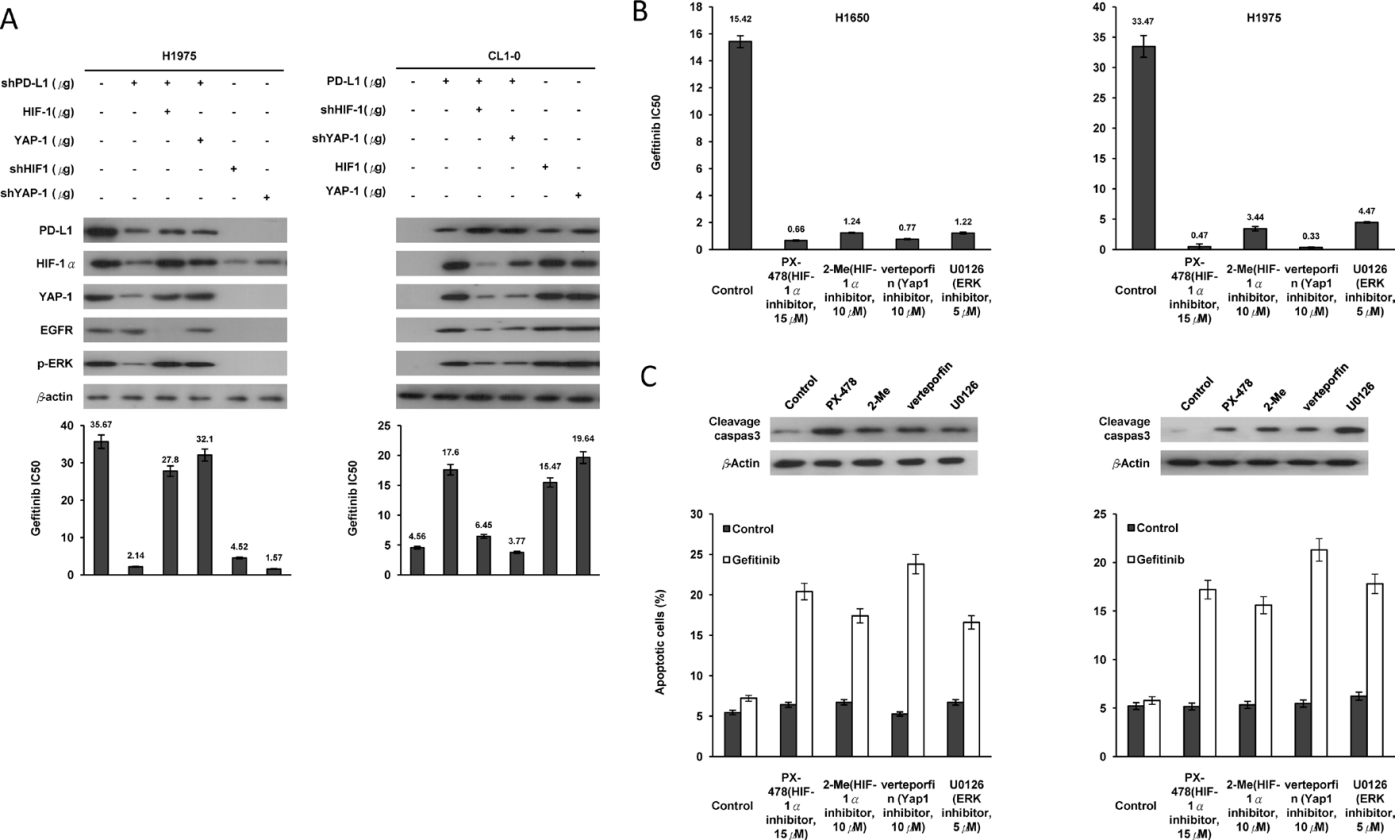
PD-L1-mediated YAP1 expression is responsible for TKI resistance in NSCLC cells (**A**) H1975 cells were transfected with PD-L1 shRNA, HIF-1 shRNA, YAP1 shRNA, HIF-1 and YAP1 expression plasmids; CL1-0 cells were transfected with PD-L1, HIF-1, YAP1 expressing plasmids, PD-L1 shRNA and HIF-1 shRNA for 48 h. The expression of PD-L1, HIF-1, and YAP1 in these cells was evaluated by western blotting using their specific antibodies. Cells were treated with six concentrations of gefitenib to calculate the IC50 value from the dose-response survival curve determined by the MTT assay. (**B**) H1650 and H1975 cell were treated with PX-478 (15 μM), 2-Me(10 μM), Verteorfin (10 μM), U0126(5 μM) and six concentrations of gefitenib to calculate the IC50 value from the dose-response survival curve determined by the MTT assay. (**C**) The expression of cleavage caspase3 in these cells was evaluated by western blotting using their specific antibodies. The cells were then subjected to annexin V and PI staining, followed by a flow cytometry. Percentage of apoptotic cells including with the Annexin V+/PI− population (early apoptosis) plus Annexin V+/PI+ (late apoptosis/secondary necrosis) was summarized by a flow cytometric analysis.

We further examined the possibility that PD-L1-mediated YAP1 expression could be responsible for EGFR mutation-independent TKI resistance in NSCLC cells. EGFR-mutated H1975 and EGFR-WT CL1-0 cells were collected for manipulation of PD-L1 expression and/or transfection with HIF-1α, a YAP1 expression plasmid, shHIF-1α, or shYAP1. The co-expression of PD-L1, HIF-1α, and YAP1 modulated by these gene manipulations was confirmed in both cell types by western blotting (Figure 3A, upper left and right panels).

The MTT assay indicated that the greatest change in the IC50 value for gefitinib was obtained with YAP1 silencing, followed, in decreasing size of the response, by PD-L1 silencing and HIF-1α silencing in H1975 cells; however, the decrease in the IC50 value obtained by PD-L1 silencing was reversed by ectopic expression of YAP1 or HIF-1α (Figure 3A lower left panel). Conversely, the IC50 value was markedly increased by ectopic expression of PD-L1, HIF-1α, or YAP1 in CL1-0 cells when compared with CL1-0 cells transfecting an empty vector (VC) (Figure 3A, lower right panel). An increase in the IC50 value by ectopic PD-L1 expression was reversed by HIF-1α or YAP1 silencing (Figure 3A, lower right panel).

A reduction in the IC50 value for gefitinib was observed in H1650 and H1975 cell types subjected to inhibitors of HIF-1α (2-methoxyestrodiol and PX478), YAP1 (verteporfin), and ERK (U0126), when compared to both cell types without these inhibitor treatments (Figure 3B upper panel). Interestingly, the expression of cleaved caspase-3, evaluated by western blotting, was increased markedly by these inhibitor treatments (Figure 3B middle panel). Annexin V-PI staining indicated that the percentage of apoptotic cells showed the greatest increase in response to verteporfin, followed, in decreasing size of the response, by PX-478, 2-methoxyestradiol, and U0126 treatment in H1650 and H1975 cells (Figure 3B lower panel). These results clearly indicated that YAP1 expression might be responsible for PD-L1-mediated TKI resistance in EGFR-mutated and –WT NSCLC cells via modulating apoptotic pathway.

### Tumor response to TKI therapy in patients with NSCLC was associated with expression of PD-L1 and YAP1

We enrolled 46 tumors from surgically resected patients with NSCLC who had received TKI therapy to examine the possibility of an association between the expression of PD-L1 and YAP1 and with tumor response to TKI therapy in these patients. All patients were adenocarcinoma and unknown EGFR mutation status. The mRNA expression levels of both genes in the lung tumor samples evaluated by real-time PCR. The median expression values for both genes then used as cutoff points to divide tumors into “high” and “low” subgroups. The PD-L1 mRNA expression level was not associated with any clinical parameters in these 46 NSCLC patients (Supplementary Table 1); however, a positive correlation was observed between PD-L1 and YAP1 mRNA expression in this study population, as high-PD-L1 mRNA tumors were frequently also high-YAP1 mRNA tumors (69.6% vs. 30.4%, *P* = 0.008; Table 1). Interestingly, tumors expressing high levels of both PD-L1 and YAP1 mRNA were more likely to show an unfavorable response to TKI therapy, when compared with tumors that expressed low levels of mRNA for both these genes (66.7% vs. 36.0%, *P* = 0.038 for PD-L1; 81.0% vs. 24.0%, P = 0.010 for YAP1; Table 2). Moreover, all patients with high-PD-L1/high-YAP1 expressing tumors exhibited an unfavorable response to TKI therapy (Table 2). These results from patients appeared to support the mechanism of action proposed for the cell model and suggested that PD-L1-mediated YAP1 expression may have the potential to predict an unfavorable response to TKI therapy in patients with NSCLC regardless of EGFR mutation.

**Table 1:** Relationships of PD-L1andYAP1 mRNA expression in NSCLC cancer patients

Characteristics	Patient No.	PD-L1		*P* value
		Low (%)	High (%)	
**Total patients**	46	23 (50.0)	23 (50.0)	
**YAP1**				
Low	23	16 (69.6)	7 (30.4)	0.008
High	23	7 (30.4)	16 (69.6)	

**Table 2:** Association of PD-L1 and YAP1 mRNA expression with tumor response to EGFR-TKI therapy in NSCLC patients

	Tumor Response				
	Patient No.	Unfavorable (%)	Favorable (%)	*P*
PD-L1				
Low	23	9 (36.0)	16 (64.0)	0.038
High	23	14 (66.7)	7 (33.3)	
YAP1				
Low	23	6 (24.0)	19 (76.0)	0.010
High	23	17 (81.0)	4 (19.0)	
PD-L1/YAP1				
Low/Low	16	6 (37.5)	10 (62.5)	0.001
Low/High	7	3 (42.9)	4 (57.1)	
High/Low	7	0 (0.0)	7 (100.0)	
High/High	16	16 (100.0)	0 (0.0)	
PD-L1/YAP1				
Others		9 (30.0)	21 (70.0)	
High/High		16 (100.0)	0 (0.0)	

PD-L1 and YAP1 mRNA levels in lung tumors were determined by real-time PCR analysis. The median value of PD-L1 (0.92) and YAP1 mRNA (1.43) in this study population was used as a cutoff point to divide patients into “low” and “high” subgroup.

Unfavorable response to TKI: patients with stable or progression disease.

Favorable response to TKI: patients with complete or partial response.

## DISCUSSION

PD-L1 is a novel direct target of HIF-1α and its blockade under hypoxia enhances MDSC-mediated T cell activation [27]. HIF-1α expression, mediated by ROS generation, plays a central role in the inflammatory tumor microenvironment [24]. Hypoxia modulates SIAH2 ubiquitin E3 ligase, which enhances the formation of a complex between YAP1 and HIF-1α, which increases HIF-1α stability [33]. Hypoxia also increases YAP1 gene expression [34]. The evidence provided here demonstrates that upregulation of YAP1 expression by PD-L1 may confer TKI resistance by increasing ROS generation and therefore promoting HIF-1α expression. This finding strongly supports our other study indicating that DDX3 confers cetuximab resistance by inducing KRAS transcription, which increases ROS-induced HIF-1α and subsequent upregulation of YAP1 expression [35]. Nevertheless, to the best of our knowledge, this is the first study to reveal transcriptional regulation of YAP1 expression by HIF-1α expression induced by PD-L1.

**Figure 4:**
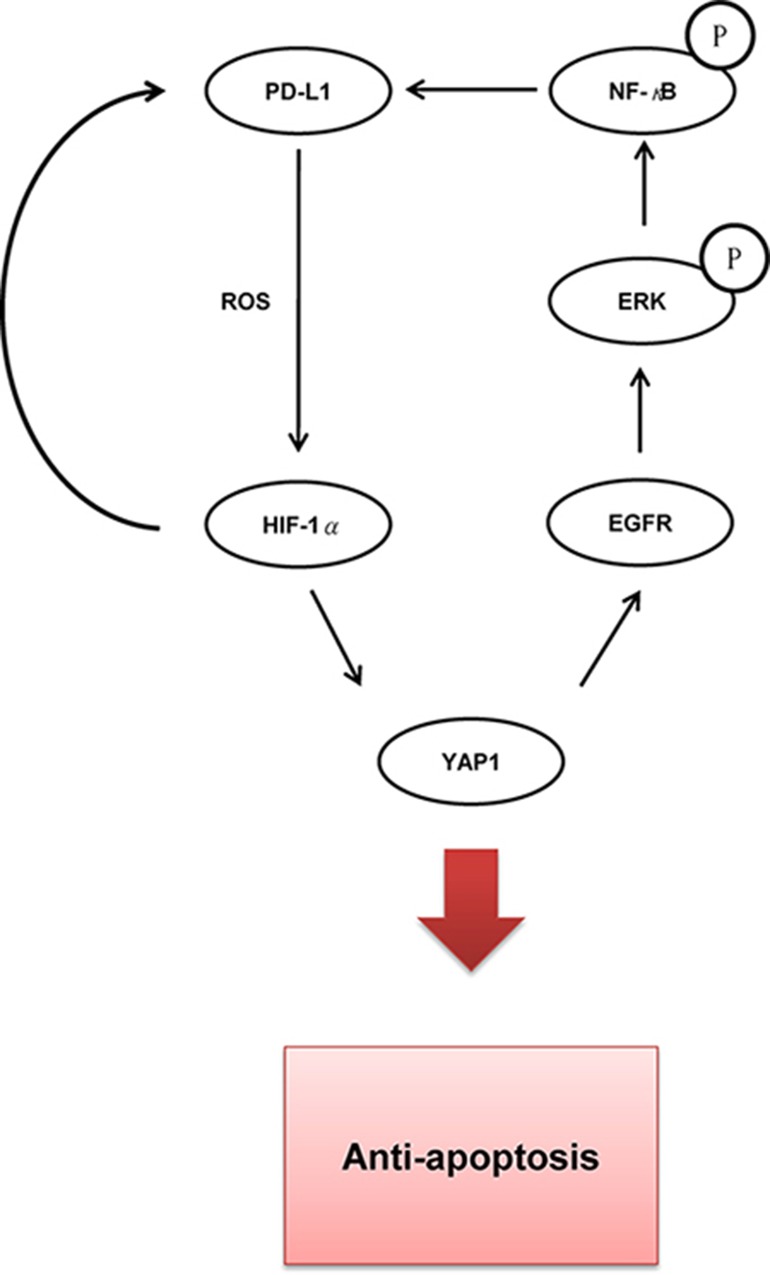
The possible route for the mechanistic action of PD-L1-induced YAP1 expression in TKI resistance of lung cancer

YAP1 is known to confer cancer drug resistance [35]. For example, YAP1 acts as a parallel survival input to promote resistance to RAF or MEK inhibitor therapy, and similarly, combined suppression of YAP and RAF or MEK enhances the treatment response and patient survival [36]. A similar finding of resistance to a MAPK inhibitor was observed in melanoma [37]. A functional genomic screen identified YAP1 as a key determinant that conferred resistance to EGFR-TKI in lung cancer cells [38]. In the present study, PD-L1 not only increased YAP1 expression, but it also elevated the expression of EGFR and p-ERK in EGFR-mutated and –WT NSCLC cells (Figures 1 and 3). A similar YAP1 upregulation of EGFR overexpression confers chemoresistance in esophageal cancer [31]. NF-κB activation may play a role in inducing PD-L1 expression and TKI resistance [15]. Our previous studies indicated that PAK1 and PD-L1 expression might confer EGFR mutation-independent TKI resistance in NSCLC via persistent activation of PI3K/AKT and MEK/ERK signaling pathways [39, 40]. TKI inhibited EGFR phosphorylation, but activation of downstream gene phosphorylation of AKT and ERK in TKI resistant EGFR-WT NSCLC cells [14]. In addition, another report indicated that NF-κB-driven suppression of FOXO3a contributed to EGFR-mutation- independent gefitinib resistance in NSCLC cells [11]. We therefore suggest that PD-L1 confers EGFR mutation-independent TKI resistance through a YAP1/EGFR/ERK/NF-κB feedback loop in NSCLC cells. A possible pathway for PD-L1-mediated TKI resistance in NSCLC cells is proposed in Figure 4.

In summary, we provided evidence that PD-L1 may confer TKI resistance in NSCLC by generating ROS to induce the expression of HIF-1α, which then upregulates YAP1 transcription. This proposed mechanism of action of PD-L1-mediated TKI resistance further validated by a retrospective study of TKI response in a small subset of NSCLC patients. Therefore, we suggest that PD-L1 immunotherapy, singly or in combination with a YAP1 inhibitor, may not only reduce tumor aggressiveness but it could also overcome TKI resistance in NSCLC, particularly in patients with tumors that express both PD-L1 and YAP1.

## MATERIALS AND METHODS

### Study subjects

Lung tumor specimens were collected from 140 patients who underwent primary NSCLC surgical resection at the Department of Thoracic Surgery, Taichung Veterans General Hospital (Taichung, Taiwan) between 1998 and 2004. Patients were asked to submit written informed consent; the study was approved by the Institutional Review Board (TMUH No. 201301051). The tumor type and stage of each collected specimen were histologically determined in accordance with the World Health Organization classification system. Among these patients, 46 patients were received TKI therapy when these patients were occurred tumor relapse. The tumor response to TKI therapy collected from chart review.

### Cell lines

TL-1 cells was kindly provided by Dr. Y.-W. Cheng (Graduate Institute of Cancer Biology and Drug Discovery, Taipei Medical University, Taipei, Taiwan) [36]. H1650, H1975, HCC827, and A549 cells were obtained from the Bioresource Collection and Research Center, the Food Industry Research and Development Institute (Hsinchu, Taiwan). CL1-0 and CL1-5 cells were kindly provided by Professor P.-C. Yang (Department of Internal Medicine, National Taiwan University Hospital, Taipei, Taiwan). TL-1, H1650, H1975, HCC827, CL1-5, and CL 1-0 cancer cell lines were maintained in RPMI-1640 medium (HyClone, Logan, UT). A549 cancer cell lines were maintained in DMEM medium (HyClone, Logan, UT). The media contained 10% fetal bovine serum (FBS) supplemented with penicillin (100 U/mL) and streptomycin (100 mg/mL). The cells were cultured according to the suppliers’ instructions. Once resuscitated, the cell lines were routinely authenticated (once every 6 months; the cells were last tested in December 2012) by cell morphology monitoring, growth curve analysis, species verification via isoenzymology and karyotyping, identity verification via short tandem repeat profiling analysis, and contamination checks.

### Real-time PCR analysis

The expression of PD-L1 mRNA and YAP1 mRNA levels in patients’ tumors was determined by real-time PCR analysis as described previously [39].

### Plasmid construction and transfection

shRNA was purchased from the National RNAi Core Facility, Academia Sinica, Taiwan. Plasmids containing the PD-L1 expression construct were constructed by cloning the full-length human PD-L1 cDNA (GenBank accession number NM_014143) into the pcDNA3.1 eukaryotic expression vector, which also expresses a neomycin (Neo) resistance gene. YAP1 and HIF1 overexpression plasmids were provided from Origene (Rockville, MD). The transfection and stable clone selection procedures have been described previously [39]. All data were collected from three independent experiments.

### 3-(4,5-cimethylthiazol-2-yl)-2,5-diphenyl tetrazolium bromide (MTT) cytotoxicity assay

The cell lines were cultured in 96-well flat-bottomed microtiter plates supplemented with RPMI 1640 and DMEM containing 10% heat-inactivated fetal bovine serum, 100 units/mL penicillin, and 100 units/mL streptomycin in a humidified atmosphere containing 95% air and 5% CO2 at 37°C in a humidified incubator. Before TKI treatment (0, 0.01, 0.1, 1, 10, 100 μM), the cells cultured in the exponential growth phase were pretreated with shRNAs, PD-L1 and BAG-1 overexpression plasmid for 24 h. After 48 h incubation, the *in vitro* cytotoxic effects of these treatments were determined by MTT assay (at 570 nm). The data were obtained from three independent experiments.

### Annexin-V/PI staining

The cells were collected by trypsinization and centrifugation at 1,000 g for 5 minutes. Following resuspension in binding buffer (10 mmol/L HEPES-NaOH, 140 mmol/L NaCl, 2.5 mmol/L CaCl_2_) at a final cell density of 1 to 2*10^6^ cells/mL, 100 μL of a single-cell suspension (1-2*10^5^ cells) was incubated with 5 μL Annexin-V–FITC and 5 μL propidium iodide (PI) for 15 minutes at room temperature in the dark. After addition of 400 Ml of binding buffer, the samples were analyzed with a BD FACS Calibur flow cytometer (BD Biosciences) within 1 hour. For each sample, 10,000 events were counted.

### Statistical analysis

Statistical analysis was calculated using by the SPSS statistical software program (Version 15.0; SPSS Inc.).

## SUPPLEMENTARY MATERIALS TABLE


