# Supplementary material for: PD-L1 confers resistance to EGFR mutation-independent tyrosine kinase inhibitors in non-small cell lung cancer via upregulation of YAP1 expression
Source: Oncotarget. 2017 Dec 8;9(4):4637–46. doi: 10.18632/oncotarget.23161 (PMC5797002; doi:10.18632/oncotarget.23161)
Supplement: Supplementary file 1 [file oncotarget-09-4637-s001.pdf]

PD-L1 confers resistance to EGFR mutation-independent tyrosine kinase inhibitors in non-small cell lung cancer via upregulation of YAP1 expression

SUPPLEMENTARY MATERIALS

Supplementary Table 1: Relationships of PD-L1expression with clinico-pathological parameters in NSCLC cancer patients

Characteristics	Patient No.	PD-L1 mRNA		*P* value
		Low (%)	High (%)	
**Total patients**	46	23 (50.0)	23 (50.0)	
Age				
≤67	24	14 (58.3)	10 (41.7)	0.238
>67	22	9 (40.9)	13 (59.1)	
**Gender**				
Female	23	11 (47.8)	12 (52.2)	0.768
Male	23	12 (52.2)	11 (47.8)	
**Smoke**				
No	36	19 (52.8)	17 (47.2)	0.475
Yes	10	4 (40.0)	6 (60.0)	
**Stage**				
I	21	10 (17.6)	11 (52.4)	0.767
II+III+IV	25	13 (52.0)	12 (48.0)	
